# mRNA-miRNA analyses reveal the involvement of CsbHLH1 and miR1446a in the regulation of caffeine biosynthesis in *Camellia sinensis*

**DOI:** 10.1093/hr/uhad282

**Published:** 2023-12-29

**Authors:** Qifang Jin, Zhong Wang, Devinder Sandhu, Lan Chen, Chenyu Shao, Fanghuizi Shang, Siyi Xie, Feiyi Huang, Zhenyan Chen, Xiangqin Zhang, Jinyu Hu, Guizhi Liu, Qin Su, Mengdi Huang, Zhonghua Liu, Jianan Huang, Na Tian, Shuoqian Liu

**Affiliations:** Key Laboratory of Tea Science of Ministry of Education, Hunan Agricultural University, Changsha 410128, China; National Research Center of Engineering and Technology for Utilization of Botanical Functional Ingredients, Hunan Agricultural University, Changsha 410128, China; CoInnovation Center of Education Ministry for Utilization of Botanical Functional Ingredients, Hunan Agricultural University, Changsha 410128, China; Key Laboratory of Tea Science of Ministry of Education, Hunan Agricultural University, Changsha 410128, China; National Research Center of Engineering and Technology for Utilization of Botanical Functional Ingredients, Hunan Agricultural University, Changsha 410128, China; CoInnovation Center of Education Ministry for Utilization of Botanical Functional Ingredients, Hunan Agricultural University, Changsha 410128, China; United States Salinity Laboratory, United States Department of Agriculture, Agricultural Research Service, Riverside, CA 92504, USA; Key Laboratory of Tea Science of Ministry of Education, Hunan Agricultural University, Changsha 410128, China; National Research Center of Engineering and Technology for Utilization of Botanical Functional Ingredients, Hunan Agricultural University, Changsha 410128, China; CoInnovation Center of Education Ministry for Utilization of Botanical Functional Ingredients, Hunan Agricultural University, Changsha 410128, China; Key Laboratory of Tea Science of Ministry of Education, Hunan Agricultural University, Changsha 410128, China; National Research Center of Engineering and Technology for Utilization of Botanical Functional Ingredients, Hunan Agricultural University, Changsha 410128, China; CoInnovation Center of Education Ministry for Utilization of Botanical Functional Ingredients, Hunan Agricultural University, Changsha 410128, China; Key Laboratory of Tea Science of Ministry of Education, Hunan Agricultural University, Changsha 410128, China; National Research Center of Engineering and Technology for Utilization of Botanical Functional Ingredients, Hunan Agricultural University, Changsha 410128, China; CoInnovation Center of Education Ministry for Utilization of Botanical Functional Ingredients, Hunan Agricultural University, Changsha 410128, China; Key Laboratory of Tea Science of Ministry of Education, Hunan Agricultural University, Changsha 410128, China; National Research Center of Engineering and Technology for Utilization of Botanical Functional Ingredients, Hunan Agricultural University, Changsha 410128, China; CoInnovation Center of Education Ministry for Utilization of Botanical Functional Ingredients, Hunan Agricultural University, Changsha 410128, China; Tea Research Institute, Hunan Academy of Agricultural Sciences/National Small and Medium Leaf Tea Plant Germplasm Resource Nursery, Changsha 410128, China; Key Laboratory of Tea Science of Ministry of Education, Hunan Agricultural University, Changsha 410128, China; National Research Center of Engineering and Technology for Utilization of Botanical Functional Ingredients, Hunan Agricultural University, Changsha 410128, China; CoInnovation Center of Education Ministry for Utilization of Botanical Functional Ingredients, Hunan Agricultural University, Changsha 410128, China; Key Laboratory of Tea Science of Ministry of Education, Hunan Agricultural University, Changsha 410128, China; National Research Center of Engineering and Technology for Utilization of Botanical Functional Ingredients, Hunan Agricultural University, Changsha 410128, China; CoInnovation Center of Education Ministry for Utilization of Botanical Functional Ingredients, Hunan Agricultural University, Changsha 410128, China; Key Laboratory of Tea Science of Ministry of Education, Hunan Agricultural University, Changsha 410128, China; National Research Center of Engineering and Technology for Utilization of Botanical Functional Ingredients, Hunan Agricultural University, Changsha 410128, China; CoInnovation Center of Education Ministry for Utilization of Botanical Functional Ingredients, Hunan Agricultural University, Changsha 410128, China; Key Laboratory of Tea Science of Ministry of Education, Hunan Agricultural University, Changsha 410128, China; National Research Center of Engineering and Technology for Utilization of Botanical Functional Ingredients, Hunan Agricultural University, Changsha 410128, China; CoInnovation Center of Education Ministry for Utilization of Botanical Functional Ingredients, Hunan Agricultural University, Changsha 410128, China; Key Laboratory of Tea Science of Ministry of Education, Hunan Agricultural University, Changsha 410128, China; National Research Center of Engineering and Technology for Utilization of Botanical Functional Ingredients, Hunan Agricultural University, Changsha 410128, China; CoInnovation Center of Education Ministry for Utilization of Botanical Functional Ingredients, Hunan Agricultural University, Changsha 410128, China; Key Laboratory of Tea Science of Ministry of Education, Hunan Agricultural University, Changsha 410128, China; National Research Center of Engineering and Technology for Utilization of Botanical Functional Ingredients, Hunan Agricultural University, Changsha 410128, China; CoInnovation Center of Education Ministry for Utilization of Botanical Functional Ingredients, Hunan Agricultural University, Changsha 410128, China; Key Laboratory of Tea Science of Ministry of Education, Hunan Agricultural University, Changsha 410128, China; National Research Center of Engineering and Technology for Utilization of Botanical Functional Ingredients, Hunan Agricultural University, Changsha 410128, China; CoInnovation Center of Education Ministry for Utilization of Botanical Functional Ingredients, Hunan Agricultural University, Changsha 410128, China; Key Laboratory of Tea Science of Ministry of Education, Hunan Agricultural University, Changsha 410128, China; National Research Center of Engineering and Technology for Utilization of Botanical Functional Ingredients, Hunan Agricultural University, Changsha 410128, China; CoInnovation Center of Education Ministry for Utilization of Botanical Functional Ingredients, Hunan Agricultural University, Changsha 410128, China; Key Laboratory of Tea Science of Ministry of Education, Hunan Agricultural University, Changsha 410128, China; National Research Center of Engineering and Technology for Utilization of Botanical Functional Ingredients, Hunan Agricultural University, Changsha 410128, China; CoInnovation Center of Education Ministry for Utilization of Botanical Functional Ingredients, Hunan Agricultural University, Changsha 410128, China; Key Laboratory of Tea Science of Ministry of Education, Hunan Agricultural University, Changsha 410128, China; National Research Center of Engineering and Technology for Utilization of Botanical Functional Ingredients, Hunan Agricultural University, Changsha 410128, China; CoInnovation Center of Education Ministry for Utilization of Botanical Functional Ingredients, Hunan Agricultural University, Changsha 410128, China

## Abstract

Caffeine, a primary flavor component in tea, has been the subject of intense research. With the goal of shedding light on the complex regulatory processes governing caffeine biosynthesis in tea plants, liquid chromatography coupled with mass spectrometry (LC–MS), transcriptomics, and small RNA analyses were employed on diverse tea cultivars such as ‘Jianghua Kucha’ [including ‘Xianghong 3’ (XH3H) and ‘Kucha 3’ (KC3H)], ‘Fuding Dabaicha’ (FDDB), ‘Yaoshan Xiulv’ (YSXL), and ‘Bixiangzao’ (BXZ). The results showed that the caffeine level in ‘Jianghua Kucha’ was significantly higher than that in other tea plant cultivars. In addition, weighted gene co-expression network analysis indicated that that the *CsbHLH1* gene might play a pivotal role as a potential hub gene related to the regulation of caffeine biosynthesis. Subcellular localization analysis showed that the CsbHLH1 protein was localized in the nucleus of the cells. Moreover, CsbHLH1 suppresses the transcription of *TCS1* by binding to the *TCS1* promoter, as evidenced by a yeast one-hybrid assay, an electrophoretic mobility shift assay, and dual luciferase analysis. In addition, a microRNA, miR1446a, was identified that directly cleaves CsbHLH1, leading to an increase in caffeine levels. Therefore, our findings imply that CsbHLH1 binds to the *TCS1* promoter (−971 to −1019 bp) to reduce its expression, thereby negatively regulating caffeine biosynthesis. On the other hand, miR1446a enhances the biosynthesis of caffeine by suppressing the expression of *CsbHLH1*. This work enhances our understanding of the molecular mechanisms of caffeine biosynthesis in tea plants and offers potential directions for manipulating caffeine levels in future tea cultivation.

## Introduction

Kucha (*Camellia sinensis*), which is mainly distributed in Hunan, Guangdong, Guangxi, and Yunnan, is a special tea resource in China [1]. When brewed, its fresh leaves offer a distinctly bitter flavor, setting it apart from other teas. Notably, ‘Jianghua Kucha’ is an excellent local landrace of Kucha recognized in Hunan Province [2], and has attracted more and more attention from researchers due to its high level of purine alkaloids compared with other tea cultivars, such as ‘Bixiangzao’ (BXZ) [2–6].

Researchers have broadly mapped out the main pathways of alkaloid biosynthesis, donor pathways, and catabolic pathways in tea plant (Supplementary Data Fig. S1). The specific biosynthesis pathway is: adenosine → guanosine → xanthosine → 7-methylxanthosine → 7-methylxanthine → theobromine → caffeine → theacrine [7–9]. Within the donor pathway, there are multiple sources for the eventual biosynthesis of xanthine nucleosides, which provide the starting methyl acceptor for the core pathway [8]. At least four pathways have been identified: the adenine nucleotide route, the guanine nucleotide route, the purine ring *de novo* biosynthesis route, and the *S*-adenosylmethionine cycle route, the adenine nucleotide route being the most critical [10–12]. Genes encoding the key enzymes in the biosynthetic pathway of alkaloids have been identified, including 7-methylxanthine synthase (catalyzing the conversion of xanthine to 7-methylxanthine), theobromine synthase (3,7-dimethylxanthine synthase, catalyzing the formation of theobromine from 7-methylxanthine), caffeine synthase (1,3,7-trimethylxanthine synthase, catalyzing the formation of caffeine from theobromine), and theacrine synthase (1,3,7,9-tetramethyluric acid synthase, catalyzing the formation of theacrine from caffeine) [13–16]. The 7-methylxanthine synthase gene (*CmXRS1*) was first cloned from coffee leaves by Mizuno *et al*. [15]. Its coding cDNA sequence is 1307 bp long and encodes a protein with 372 amino acids. Suzuki and Takahashi isolated and identified theobromine synthase and caffeine synthase from crude tea extracts for the first time [17]. The *CgcTS* gene, which encodes theobromine synthase, was cloned from young leaves of the tea plant *Camellia gymnogyna* Chang of Danyao Mountain, Guangxi [16]. Additionally, the *TCS1* gene, which has a 1483-bp open reading frame encoding 369 amino acids, was first cloned from tea shoots [18]. Recently, theacrine synthase CkTcS1 isolated from Kucha was identified as an *N*-methyltransferase with novel activity, which uses theacrine as substrate instead of caffeine, indicating that C8 oxidation occurs prior to *N*9 methylation [19].

Although the biosynthetic pathway of caffeine has been elucidated and the key enzymes and their coding genes involved in the caffeine biosynthetic pathway have been identified, the molecular regulation of caffeine biosynthesis is still unclear [20–23]. So far, only a few transcription factors (TFs) related to the regulation of caffeine levels in plants have been discovered. MYB184 has been reported to bind and activate the TCS1 promoter, as confirmed by yeast single-hybridization experiments and transactivation assays [24]. The binding of MYB1AT to the fused MYBPLANT motif was further validated *in vitro* by an electrophoretic mobility shift assay (EMSA), which established MYB184 as a key activating agent of *TCS1*, and the production of caffeine in tea [24].

The mechanism and function of microRNA (miRNA) in the secondary metabolism of the tea plant has gradually developed into a new direction of modern tea plant research [25–28]. At present, most of the studies on the regulation of caffeine biosynthesis are from the perspective of structural genes and TFs, and fewer studies have been reported on the involvement of miRNAs in caffeine biosynthesis in the tea plant. miRNA serves as a key inhibitor in gene expression, primarily managing gene methylation during transcription and guiding mRNA translation in the post-transcription phase to control gene expression [29–30]. Its role extends to complex biological functions, regulating numerous target mRNA molecules. In plants, miRNA is integral in shaping plant morphology, facilitating signal transduction, orchestrating hormonal responses, enhancing stress tolerance, overseeing nutrient metabolism, and playing a vital part in various facets of plant growth and development [26–35]. The miRNAs have been shown to negatively regulate catechin biosynthesis through the downregulation of their biosynthesis-related target genes [36]. Epigallocatechin gallate from green tea suppresses the growth of 3T3-L1 cells through the miRNA-143/MAPK7 pathways [34]. Melatonin significantly increased transcript levels of several miRNAs, including miR-171b. Importantly, the α-glucan water dikinase (*GWD*) gene was identified as miR171b’s target [37]. Chlorophyll and starch degradation were improved and *GWD* gene expression was suppressed by miR171b overexpression [37]. These results suggest that melatonin may be able to delay dark-induced leaf senescence in tomato by inducing the expression of miR171b. In another study, miR319b was shown to target *CsTCP3* transcription to control the development of the shoot tip and the biosynthesis of catechins in the tea plant [38]. Moreover, miRNAs were found to cross-regulate *N*-methyltransferase genes in *Coffea canephora*, which are involved in the biosynthesis of caffeine [25]. Nonetheless, there is a notable scarcity of research focusing on the pathway of caffeine biosynthesis in the tea plant, specifically concerning the regulatory role of miRNA in caffeine biosynthesis. This area merits further exploration and study.

In our study, we identified the *CsbHLH1* as a possible central gene involved in the biosynthesis of the purine alkaloids of the tea plant. Yeast one-hybrid assays, EMSA assays, antisense oligonucleotide (As ODN) assays, and dual luciferase reporter gene assays have shown that CsbHLH1 can bind to the *TCS1* promoter, inhibit the transcription of the initiating *TCS1*, and reduce caffeine content in tea plant shoots. The 5′-RLM–RACE (RNA ligase-mediated rapid amplification of cDNA ends) assay showed that miR1446a could positively regulate *TCS1* biosynthesis by repressing the target gene *CsbHLH1*.

## Results

### Analysis of the main alkaloid contents of different tea plant cultivars

**Figure 1:**
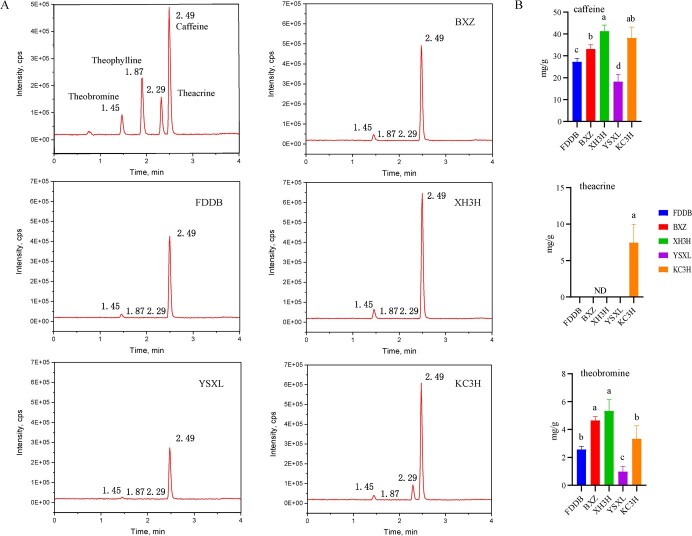
High performance liquid chromatograms of standards and major alkaloids of five tea plant cultivars. **A** Standard. **B** Chromatograms of the main purine alkaloid substances of BXZ. **C** Chromatograms of the main purine alkaloid substances of FDDB. **D** Chromatograms of the main purine alkaloid substances of KC3H. **E** Chromatograms of the main purine alkaloid substances of XH3H. **F** Chromatograms of the main purine alkaloid substances of YSXL. **G** Main alkaloid components of different tea plant cultivars. The data represent the mean from three replicates. ND, not detected. Different small letters indicate a significant different difference among different treatments (*P* < 0.05).

The results of the detection of the main alkaloid substances of the five different tea plant cultivars are shown in Fig. 1. Compared with the tea plant cultivars ‘Fuding Dabaicha’ (FDDB), ‘Yaoshan Xiulv’ (YSXL) and BXZ, ‘Jianghua Kucha’ cultivars [‘Xianghong 3’ (XH3H) and ‘Kucha 3’ (KC3H)] were significantly higher in caffeine content than the conventional tea plant cultivars, with XH3H having the highest caffeine content, 41.3 mg g^−1^ (Fig. 1G). YSXL had the lowest caffeine content. The highest levels of theobromine were found in XH3H (one of the ‘Jianghua Kucha’ cultivars), while KC3H (another ‘Jianghua Kucha’ cultivar) displayed the highest level of theacrine (Fig. 1G). It is worth noting that theacrine was not detected in any of the cultivars except KC3H, which contained 7.5 mg g^−1^. This indicates that there is a large difference in alkaloid composition between ‘Jianghua Kucha’ and conventional tea plant cultivars.

### Transcriptomic data analysis and differentially expressed gene identification and validation

Sequencing of 15 tea samples yielded an average of 6.39 Gb of high-quality data for all cDNA libraries, characterized by an average Q30 level of 97.36%, indicating a high degree of accuracy (Supplementary Data Table S1). The read mapping ratio to the reference genome ranged from 82.47 to 85.09% (Supplementary Data Table S2) for each sample. The values of the coefficient of determination for the FPKM (fragments per kilobase million) between biological duplicates were 0.96 ≤ *R*^2^ ≤ 0.99, indicating a good degree of reproducibility (Supplementary Data Fig. S2A).

In addition, 48 984 unique genes were found, of which 35 273 were differentially expressed in the 10 groups (Supplementary Data Fig. S2B). Twelve differentially expressed genes (DEGs) were selected for real-time qRT–PCR analysis aimed at validating the different patterns of expression that were observed in the RNA-seq data (Supplementary Data Table S3). We found that changes in expression levels of those obtained from the RNA-seq data were consistent with changes in expression levels from qRT–PCR (Supplementary Data Fig. S3). These results reinforced the reliability of the RNA-seq data obtained in this study.

### GO and KEGG enrichment analysis of the differentially expressed genes

**Figure 2:**
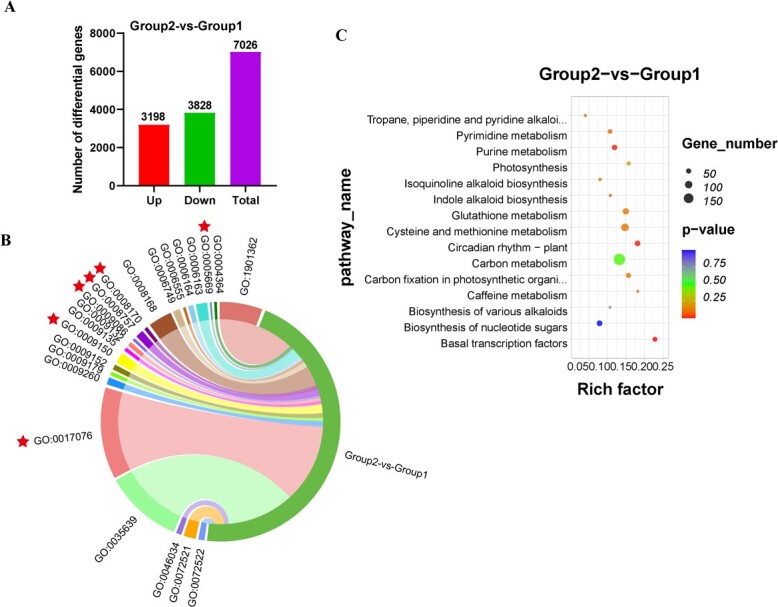
Pairwise comparisons of GO and KEGG enrichment analysis. **A** Analysis of DEGs in group 2 versus group 1. **B** Enrichment GO terms of DEGs from group 2 versus group 1. **C** KEGG enrichment terms of DEGs from group 2 versus group 1.

In order to further reveal the DEGs specific to ‘Jianghua Kucha’ cultivars (group 1: XH3H and KC3H) and conventional tea plant cultivars (group 2: FDDB, BXZ, and YSXL), comparative analyses of the DEGs between the two groups of tea plant cultivars were carried out. A total of 7026 DEGs were screened in group 2 versus group 1. Among them, 3198 were upregulated and 3828 were downregulated (Fig. 2A). The DEGs of group 2 versus group 1 mapped to 4141 GO terms (Supplementary Data Table S4). Specifically with this comparison, we found 111 GO terms that are potentially associated with caffeine biosynthesis. Of these identified GO terms, 81 were characterized under the biological process (BP) category, 7 under cellular components (CC), and 23 were associated with molecular functions (MF) (Supplementary Data Table S5). These GO terms are mainly related to TFs, purine ribonucleotide metabolic process, methionine biosynthetic process, purine nucleotide binding, *S*-adenosylmethionine-dependent methyltransferase activity, and *N*-methyltransferase activity (Supplementary Data Table S6; Fig. 2B). In addition, the group 2 versus group 1 comparator group was enriched for 139 pathways according to KEGG analysis (Supplementary Data Table S7). Notably, a number of pathways, such as carbon metabolism (ko01200), purine metabolism (ko00230), photosynthetic biocarbon fixation (ko00710), circadian-plant (ko04712), pyrimidine metabolism (ko00240), cysteine and methionine metabolism (ko00270), biosynthesis of various alkaloids (ko00996), tropane, piperidine and pyridine alkaloid biosynthesis (ko00960), and caffeine metabolism (ko00232) are related to caffeine biosynthesis (Fig. 2C). Purine and caffeine metabolism pathways are essential for the production of caffeine alkaloids, and the data showed that 57 DEGs were enriched in purine metabolism in the group 2 versus group 1 comparator group. Additionally, two DEGs were notably enriched in caffeine metabolism (Supplementary Data Table S8). We found that the major differential GO terms and KEEG pathways enriched were broadly consistent regardless of the comparison method.

### Differentially expressed genes involved in biosynthesis of purine alkaloids

Since the purine alkaloid content of ‘Jianghua Kucha’ tea differs from that of conventional tea cultivars, we then performed transcriptomic data analysis on these samples to look at the gene expression associated with them. Theobromine synthases (3-NMTs), caffeine synthases (TCS1), and theacrine synthases (9-NMTs) catalyze the last three *N*-methylations in the purine alkaloid biosynthetic pathway, respectively. Compared with other enzymes associated with this pathway, they are considered to be three key enzymes and have been extensively studied.

**Figure 3:**
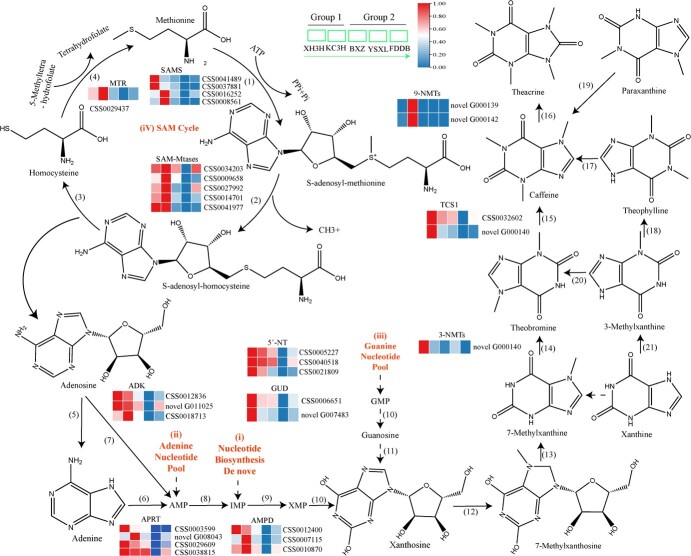
**Biosynthesis pathways of purine alkaloids and expression of related genes.** Biosynthesis pathways of purine alkaloids and expression of related genes. (1) SAMS: *S*-adenosine-l-methionine synthetase. (2) SAM-Mtases: *S*-adenosine- l-methionine dependent methyltransferase. (3) SAH hydrolase: *S*-adenosine-l-homocysteine hydrolase. (4) MTR: methionine synthase. (5) Adenosine nucleosidase. (6) APRT: adenine phosphoribosyl transferase. (7) ADK: adenosine kinase. (8) AMPD: adenosine 5′-monophosphate deaminase. (9) Inosine 5′-monophosphate dehydrogenase. (10) 5′-NT: 5′-nucleotidase. (11) GUD: guanosine deaminase. (12) 7-Methylxanthosine synthase. (13) 7-Methylxanthosine nucleosidase. (14) 3-NMTs: theobromine synthase. (15) TCS1: caffeine synthase. (16) 9-NMTs: theacrine synthases. (17) 7-Methyltransferase. (18) Theophylline synthetase. (19) 3-Methyltransferase. (20) 7-Methyltransferase. (21) 3-Methylxanthine synthetase. Group 1: ‘Jianghua Kucha’; group 2: conventional tea plant cultivars.

Expression levels of *3-NMT*s and *TCS1* in new shoots of KC3H and XH3H tea plants were much higher than those of YSXL (Fig. 3). The expression of *3-NMT* and *TCS1* genes followed the same trend as the metabolite content. Except for KC3H, transcription levels of two *9-NMT* genes were zero or very low in the shoots of all other tea plant cultivars (Fig. 3). These results indicated that the alkaloid contents of five tea cultivars were consistent with the expression patterns of most *3-NMT*s, *9-NMT*s, and *TCS1*. Therefore, according to metabolite analysis and expression patterns of three key genes, *3-NMT*s, *9-NMT*s, and *TCS1* positively regulate the biosynthesis of purine alkaloids.

Several purine precursors of the purine alkaloids have been suggested (Fig. 3). SAMS catalyzes the conversion of methionine to *S*-adenosine-l-methionine (SAM). SAM is an important methyl donor in the biosynthesis of caffeine methylation. The expressions of *SAMS* (CSS0041489) and *SAMS* (CSS0037881) were the highest in the tender shoot of XH3H. The expressions of *SAMS* (CSS0016252) and *SAMS* (CSS0008561) were the highest in the tender shoot of KC3H (Fig. 3). *S*-Adenosine-l-methionine-dependent methyltransferases (SAM-Mtases) catalyze the conversion of SAM to *S*-adenosine homocysteine. The expressions of five *SAM-Mtase*s, namely CSS0041977, CSS0034203, CSS0009658, CSS0027992, and CSS0014701 were the highest in KC3H (Fig. 3). The conversion of SAH to homocysteine and adenosine is catalyzed by *S*-adenosine-l-homocysteine hydrolase (SAHH). There was no difference in the expression of the *SAHH* gene among the five tea cultivars in this study. Adenosine kinases (ADKs) are catalysts for converting adenosine to adenosine 5′-monophosphate. Among these, *ADK* (CSS0018713) showed maximum expression in the KC3H variety, while *ADK* (CSS0012836) and *ADK* (novel G011025) were more prominently expressed in XH3H (Fig. 3). Adenosine riboside catalyzes the conversion of adenosine to adenine, and adenine phosphoribosyl transferase (APRT) catalyzes the conversion of adenine to adenosine 5′-monophosphate (AMP). All four *APRT* genes, novel_G008043, CSS0003599, CSS0029609, and CSS0038815, displayed the highest expression in one of the ‘Jianghua Kucha’ cultivars: XH3H or KC3H (Fig. 3). Furthermore, *IMPDH*, encoding inosine 5'-monophosphate dehydrogenase (IMPDH), catalyzes the conversion of inosine 5′-monophosphate (IMP) to xanthosine 5′-monophosphate (XMP). The 5′ nucleotide enzyme catalyzes the conversion of XMP to xanthosine. However, there was no difference in the gene expression level of *IMPDH*, among the five tea cultivars in this study. The 5′-nucleotide (5′-NT) is responsible for the conversion of guanosine 5′-monophosphate (GMP) to guanosine. The expression levels of *5′-NT* and *GUD* genes were the highest in XH3H, followed by KC3H (Fig. 3).

**Figure 4:**
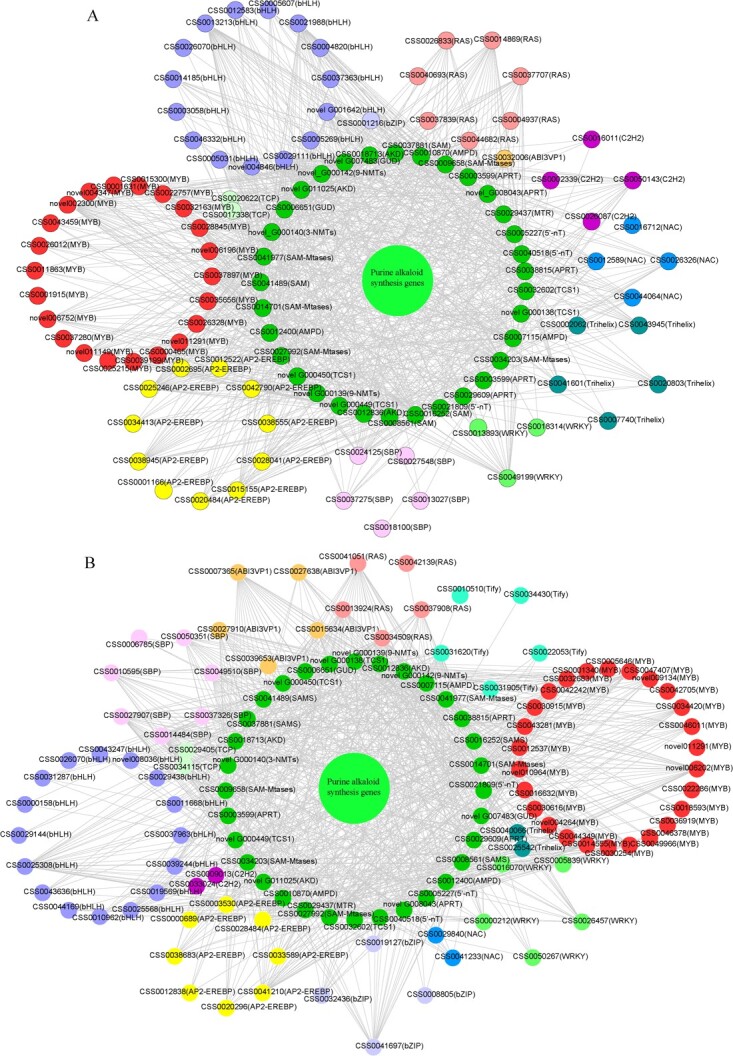
All identified TFs in the TF-mRNA network. **A** Network interaction of key genes of purine alkaloid biosynthesis positively correlated with TFs (*R*^2^ > 0.65). **B** Network interaction of key genes of purine alkaloid biosynthesis negatively correlated with TFs (*R*^2^ > 0.65).

TFs have been considered to play a crucial role in the regulation of the biosynthesis of purine alkaloids in tea plants. To further understand the potential function of these TFs in purine alkaloid biosynthesis in tea plants, we systematically analyzed DEGs encoding TFs. We identified a total of 213 differentially expressed TFs from 13 different families, which included MYB, bHLH, AP2-EREBP, RAS, SBP, NAC, Trihelix, WRKY, TCP, ABI3VP1, bZIP, C2H2, and Tify (Supplementary Data Table S9). Correlation values were calculated using the R language based on FPKM values of 213 TFs and key genes for purine alkaloid biosynthesis. We specifically focused on TFs with correlation coefficients >0.65 (Supplementary Data Table S9). Cytoscape software was used to draw positive and negative correlation network interactions between TFs and key genes for purine alkaloid biosynthesis (Fig. 4). The TFs showing the most interactions belonged to the bHLH, AP2-EREBP, MYB, RAS, SBP, and ABI3VP1 families (Supplementary Data Table S9). Based on these findings, we speculate that the biosynthesis of alkaloids may involve these TFs.

### Co-expressed genes related to purine alkaloid content

**Figure 5:**
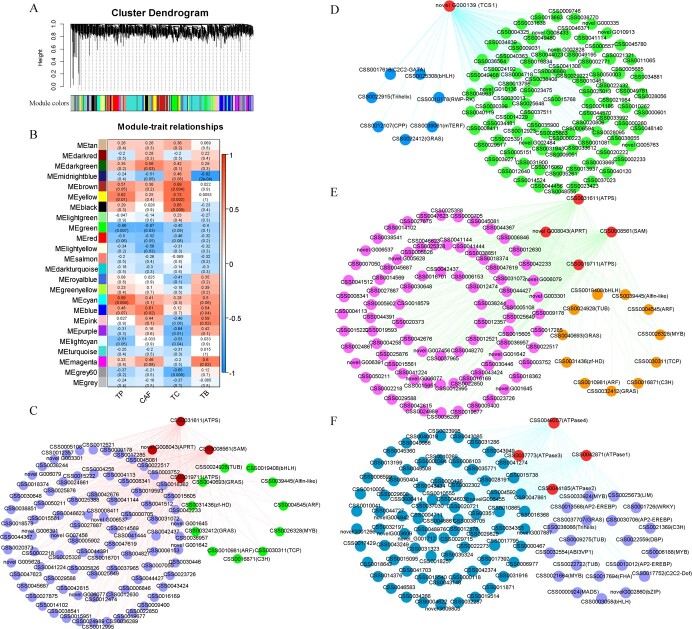
Co-expression network analysis of tea plant purine alkaloids. **A** WGCNA analysis-based hierarchical clustering tree of modules. **B** Relationships between modules and the characteristics of the samples tested. The numbers represent R and the numbers in parenthesis represent *P* values. **C** Co-expression network diagram of candidate genes in the ME magenta module. **D** Candidate gene co-expression network diagram in the ME green module. (E) ME brown module co-expression network diagram of candidate genes. **F** Co-expression network diagram of the candidate genes in the ME cyan module.

The application of weighted gene co-expression network analysis (WGCNA) enabled us to identify gene sets associated with purine alkaloid traits (theobromine (TB), caffeine (CAF), theacrine (TC), and Theophylline (TP)) in tea plants. This analysis used a dataset of 35 273 DEGs and associated phenotypes. Twenty-five different co-expression modules (marked by 25 different colors; gray modules are sets of genes not assigned to other modules) of WGCNA were produced, containing closely connected gene clusters with high correlation coefficients between genes in the same cluster (Fig. 5A). The turquoise module aggregated the most genes, with 5599 genes, and saddle brown had only 92 genes, averaging 1310 genes per module (Fig. 5B). The ME magenta module (682) was strongly correlated with TB (*r* = 0.6, *P* = 0.02), the ME brown module (1098) was significantly and positively correlated with TC (*r* = 0.69, *P* = 0.004), and the ME cyan module (390) with TP (*r* = 0.69, *P* = 0.004) (Fig. 5B). In addition, the ME green module (980) was significantly negatively correlated with CAF (*r* = −0.67, *P* = 0.03) and the ME midnight blue module (372) with TB (*r* = −0.82, *P* = 2e^−04^) (Fig. 5B). These results suggest that the genes most likely to regulate purine alkaloid formation in the tea plant are those belonging to these five modules.

In addition, we selected the TFs and purine biosynthesis-related genes as key hub genes using the top 100 unigene pairs in the magenta, blue, brown, and cyan modules to construct the co-expression network. In all, 15 key hub genes have been identified, which include two *ARF*, two *GRAS*, *bHLH*, *C3H*, *MYB*, *TUB*, *Alfin-like*, *zf-HD*, and *TCP* TF genes in the ME magenta module (Fig. 5C). Additionally, we identified three pivotal genes (*APRT*, *ATPS*, and *SAM*) that play a central role in the purine alkaloid biosynthesis pathway (Fig. 5C). Eight genes were selected as key hub genes for the green ME module. Of these, seven were TF genes, including one *C2C2-GATA*, one *Trihelix*, one *CPP*, one *GRAS*, one *mTERF*, one *RWP-RK*, and one *bHLH* (Fig. 5D). In addition, there was one *TCS* gene in the purine alkaloid biosynthesis pathway (Fig. 5D). A total of 15 key hub genes were selected for the ME brown module. Of these, 11 were TFs. These included two *GRAS*, two *ARF*, one *bHLH*, one *zf-HD*, one *TUB*, one *C3H*, one *TCP*, one *MYB*, and one *Alfin-like* gene (Fig. 5E). In addition, two *ATPS* genes, one *APRT* gene, and one *SAM* gene were identified in the purine alkaloid biosynthesis pathway (Fig. 5E). In the cyan module of the ME, 24 genes were selected as the most important hub genes. Of these, 20 were TF genes. These included three *AP2-EREBP*, three *MYB*, two *ABI3VP1*, two *TUB*, one *WRKY*, one *bHLH*, one *bZIP*, one *Trihelix*, one *C3H*, one *C2C2-Dof*, one *DBP*, one *LIM*, one *MADS*, one *FHA*, and one *GRAS* (Fig. 5F). In addition, four *ATPase* genes related to the purine alkaloid biosynthesis pathway were included (Fig. 5F). These analyses suggest that the molecular regulatory mechanism of purine alkaloid formation in the tea plant is complex, involving many different functional genes related to purine alkaloid biosynthesis, such as the TF family.

### Interaction analysis of miRNAs and target genes

**Figure 6:**
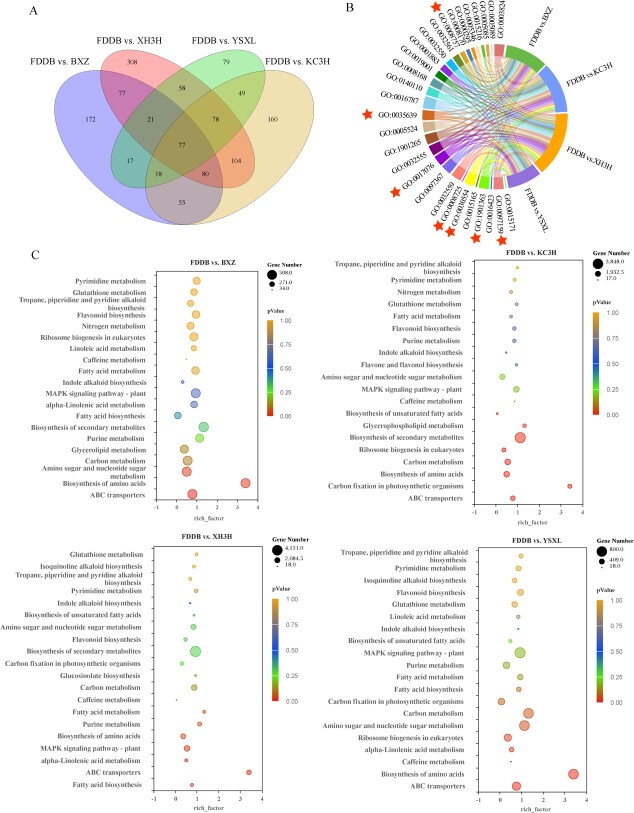
Pairwise comparisons of differential gene GO and KEGG enrichment analysis of miRNAs. **A** Venn diagram of co-expressed DEGs and uniquely expressed DEGs for the four comparisons. **B** Overall enrichment terms of DEGs of FDDB versus XH3H, FDDB versus KC3H, FDDB versus YSXL, and FDDB versus BXZ comparisons. **C** KEGG enrichment terms of DEGs of FDDB versus XH3H, FDDB versus KC3H, FDDB versus YSXL, and FDDB versus BXZ comparisons.

In total, 123.08 Gb of clean tags were obtained from the five tea samples. After filtering, the total clean reads of each sample in this project were from 20.95 to 23.88 M. The genome mapping rate ranged from 86.92 to 89.22%. The sncRNA mapping rate accounted for 11.49–8.65%. Non-coding RNAs were identified as snoRNA (406), rRNA (321), sRNA (237), cis-reg (199), tRNA (94), lncRNA (66), CRISPR (59), Rfam_other_RNA (50), ribozyme (17), antisense (15), other (15), snRNA (12), antitoxin (6), misc_RNA (4), the predicted non-coding RNA as miRNA (6422), and siRNA (1015). In addition, 77 differential genes were common to the four comparator groups (Fig. 6A). Results of GO enrichment analysis showed that DEGs were mainly enriched in methyltransferase activity, purine nucleotide binding, *S*-adenosylmethionine-dependent methyltransferase activity, *N*-methyltransferase activity, and carbohydrate derivative binding in the four comparator groups (Fig. 6B). KEGG enrichment analysis showed that the main metabolic pathways enriched for differential genes in the four comparison groups were carbon metabolism, MAPK signaling pathway-vegetation, purine metabolism, glutathione metabolism, pyrimidine metabolism, scopolamine, caffeine metabolism, and indole alkaloid biosynthesis (Fig. 6C).

In addition, KEGG enrichment analysis allowed the identification of 140, 137, 137, and 138 metabolic pathways for the four comparator groups. Of these, four pathways, ko00270, ko00230, ko00232, and ko00480, were associated with purine alkaloid biosynthesis, comprising 870, 909, 979, and 839 differentially expressed target genes, respectively (Supplementary Data Fig. S4A). Analysis of differentially expressed target genes and miRNA interactions in the purine alkaloid biosynthesis pathway further showed that 203 miRNAs were localized to the comparator group, with 36, 42, 17, and 27 upregulating gene expression and 48, 60, 29, and 35 downregulating gene expression, respectively (Supplementary Data Fig. S4B). The correlation analysis between target genes in the transcriptome and DEGs suggested that the *TCS1* gene (CSS0032602.3) associated with caffeine biosynthesis may be under the regulatory control of several miRNAs. These include novel-miRNA-2129-5p, novel-miRNA-2130-5p, novel-miRNA-1821-3p, novel-miRNA-1822-3p, novel-miRNA-1823-3p, novel-miRNA-2083-3p, and novel-miRNA-849-5p. These miRNAs have been identified as primary candidates for involvement in the regulation of purine alkaloids (Supplementary Data Fig. S4C and Supplementary Data Table S10).

In the above study, we calculated correlation values based on the FPKM values of 213 TFs and key genes for purine alkaloid biosynthesis. A total of 167 TFs with correlation coefficients >0.65 were screened. Among these, the TF CsbHLH1 (CSS0025308) showed a strong correlation, ranging from −0.97 to −0.65, with 10 critical genes involved in purine alkaloid biosynthesis (Supplementary Data Table S9). Based on 35 273 DEGs and phenotypic data, WGCNA captured the set of genes associated with purine alkaloid traits (TB, CAF, TC, and TP) in the tea plant. The correlation coefficient between CsbHLH1 and novel G000139 (TCS) in the green module was −0.87. In addition, using the JASPAR in-line software for promoter binding prediction, we found that the bHLH TF CsbHLH1 was able to bind to the *TCS1* promoter, scoring 10, the highest score among all bHLH TFs predicted to bind the same promoter. Thus, CsbHLH1 was chosen for further investigation.

### Subcellular localization analysis of CsbHLH1

**Figure 7:**
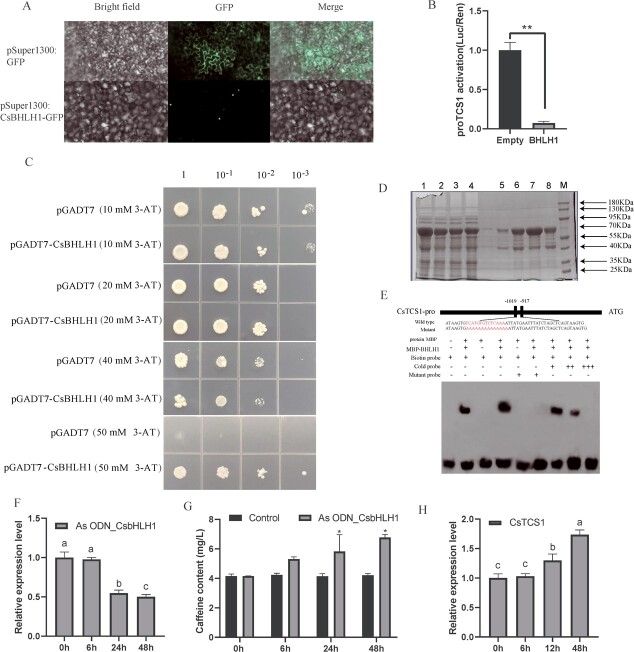
CsbHLH1 is localized in the nucleus of the cell and has transactivating activity, and it binds to the promoter of *CsTCS1*. **A** Subcellular location of CsbHLH1 in tobacco leaf; scale bars = 10 μm. **B** Transcriptional transactivation in tobacco. **C** Transcriptional activity of CsbHLH1 in yeast cells. **D** Protein purification to obtain a fusion protein of MBP-CsbHLH1. M, protein marker; 1 and 2, supernatant and precipitation of 0.5 mmol/l IPTG-induced protein; 3 and 4, supernatant and precipitation of 0.7 mmol/l IPTG-induced protein; 5, 6, and 8, purified CsbHLH1 recombinant protein washing solution. 7, concentrated CsbHLH1 recombinant protein washing solution. **E** EMSA. From left to right: negative control; positive control; CsTCS1 probe; CsTCS1 probe bound to CsbHLH1 protein; CsTCS1 mutant probe; CsTCS1 mutant probe and CsbHLH1 protein; CsbHLH1, CsTCS1 mutant probe, and 10× CsTCS1 competitive probe; CsTCS1 mutant probe and 50× CsTCS1 competitive probe; CsTCS1 mutant probe and 100× CsTCS1 competitive probe. The 10× and 50× CsTCS1 competitive probes can compete with part of the strip, and the 100× CsTCS1 competitive probes can compete with all the strip. **F** Gene expression of CsbHLH1 in leaves after As ODN_CsbHLH1 treatment expression. **G **Caffeine content in young shoots treated with water and As ODN_CsbHLH1. **H** Relative expression of *CsTCS1* after inhibition of *CsbHLH1*.

In order to determine the subcellular localization of CsbHLH1, pSuper1300-CsbHLH1-GFP was transiently expressed in tobacco leaves, while pSuper1300-GFP was used as a control in the epidermal cells of tobacco leaves. The results indicated that CsbHLH1 was located in the nucleus (Fig. 7A).

### CsbHLH1 represses transcription of the *TCS1* promoter

CsbHLH1 transcription was assessed using a double luciferase reporter gene analysis system. The results showed that the LUC/REN values of the reporter gene co-injected with pGreenII 62-SK-CsbHLH1 were significantly (*P* < 0.05) lower than those of the negative control (pGreenII 62-SK-Empty) (Fig. 7B). This indicates that the CsbHLH1 protein is able to inhibit the transcription of the *TCS1* promoter.

### Yeast one hybrid assays with CsbHLH1

To further investigate the transcriptional activating activity of CsbHLH1, recombinant plasmids containing the corresponding sequences were transferred to yeast monohybrid (Y187) receptor cells and grown in the appropriate media. pHis2-bait plasmids were obtained by constructing the *TCS1* promoter (−953 to −1556 bp) into the pHis2 vector and the complete coding sequence of CsbHLH1 was constructed into the pGADT7 vector to obtain the AD-prey plasmid. pHis2-bait and AD-prey plasmids were mixed as the experimental group, and pHis2-bait and empty pGADT7 plasmids were mixed as the self-activation group. On the SD/−His/−Leu/−Trp medium, the growth of the Y187 strain transformed by the recombined vectors harboring the promoters of either *TCS1* was inhibited by 50 mM 3-amino-1,2,4-trisole (3-AT). After transformation with the prey vector pGADT7-CsbHLH1, the strains with TCS1 grew and produced few colonies compared with the pGADT7 empty vector control on the SD/−His/−Leu/−Trp plates containing 50 mM 3-AT at the same dilution (Fig. 7C).

### Electrophoretic mobility shift analysis of the *TCS1* promoter

The fusion protein MBP-CsbHLH1 was obtained by IPTG (isopropyl-d-1-thiogalactopyranoside) induction and protein purification, setting the stage for the EMSA assay (Fig. 7D). The promoter sequence of *TCS1* (−971 bp to −1019 bp) contains a predicted MYC binding site (CATGTG) and the EMSA results showed that CsbHLH1 was able to bind to the MYC binding site (CATGTG) in the *TCS1* promoter (Fig. 7E). The mutation probe was synthesized by mutating the sequence of the MYC site (CATGTG) in its entirety to the A base, ultimately identifying the *TCS1* promoter binding region.

### Impact of inhibition of *CsbHLH1* on caffeine biosynthesis

The oligodeoxynucleotide As ODN_CsbHLH1, designed to target and suppress the expression of *CsbHLH1*, proved effective in achieving significant inhibition after 24 and 48 h in samples placed in centrifuge tubes (Fig. 7F). This is evidence of the effective inhibitory capability of As ODN_CsbHLH1. Furthermore, we observed a notable increase in the caffeine content in the tea plant shoots treated with As ODN_CsbHLH1 at the 24- and 48-h marks, compared with the control group treated with water (Fig. 7G). This suggests that the suppression of *CsbHLH1* gene expression is directly associated with an uptick in caffeine concentration.

### Characterization of miR1446a target gene

*CsbHLH1* was predicted using psRNATarget software based on miRNA information in tea leaves obtained by bioinformatics and high-throughput sequencing. Thirty-three miRNAs were predicted to be able to cleave *CsbHLH1* (Supplementary Data Table S11). Cleavage site validation was performed using modified RLM–RACE. Our findings unequivocally demonstrate that miR1446a specifically targets and cleaves the downstream TF CsbHLH1, an integral component in the regulation of caffeine biosynthesis (Supplementary Data Fig. S5). This key observation provides compelling evidence for the suppressive role of miR1446a in the regulation of *CsbHLH1* expression.

## Discussion

In the tea plant, the primary purine alkaloids include caffeine, theobromine, theophylline, and theacrine, caffeine being the most prevalent [4]. Typically, caffeine accounts for 2–5% of the dry matter content, making it a key quality and functional component of tea [39]. Consequently, the process of caffeine biosynthesis has garnered significant attention [6]. While the pathway of caffeine biosynthesis is now largely understood, the details of its regulatory mechanisms still require further exploration and study.

In order to reveal the transcriptional regulation mechanism of caffeine in tea, we obtained five tea plant cultivars with gradient changes in caffeine level (ranging from 41.3 to 18.1 mg g^−1^), selected from a pool of >50 different resources. The five chosen tea cultivars served as excellent resources for isolating the key regulatory genes. This was achieved by analyzing the relationship between caffeine content and gene expression levels using transcriptome data and WGCNA. WGCNA has been widely used in mining transcriptionally regulated genes for secondary metabolite biosynthesis [40–42]. It also helps to identify highly related gene clusters and reveal their regulatory networks in plant secondary metabolite biosynthesis. In a previous study, three modules related to marker metabolites were identified, and key structural genes potentially influencing hypoxic germination were pinpointed from these modules through a WGCNA analysis of targeted metabolomics and transcriptomics [43]. Recently, the use of the WGCNA approach to establish a link between tea aroma accumulation and gene expression revealed 13 TF genes that may be involved in terpene metabolism [44]. Among these genes, *CsOCS2* was verified *in vitro* to play a role in terpene biosynthesis, indicating the reliability of WGCNA for trait-related gene discovery [44]. In this study, our transcriptome analyses revealed a greater GO enrichment of methionine biosynthetic process, *S*-adenosylmethionine-dependent methyltransferase activity, and *N*-methyltransferase activity in ‘Jianghua Kucha’ cultivars compared with conventional tea cultivars (Fig. 2B and Supplementary Data Table S6). Furthermore, the KEGG pathways were more enriched in purine and caffeine metabolisms (Fig. 2C and Supplementary Data Table S8). The expression pattern of *CsbHLH1* was negatively correlated with the expression pattern of *TCS1* and showed an opposite trend to the caffeine content (Figs 1G and4B and Supplementary Data Table S9). Based on WGCNA analysis, we found a novel TF, CsbHLH1, which regulates the gene expression of *TCS1* (Fig. 5).

The bHLH TFs, which have been identified across a wide range of cultivars, hold a crucial place in the biosynthetic regulation of plant polyphenols [45–50]. The overexpression of *PabHLH* in both callus and thallus boosted bisbibenzyl accumulation and upregulated *PaPAL*, *Pa4CL1*, *PaSTCS11*, and two P450 cytochrome-encoding genes [54]. The bHLH-related TF DcTT8, isolated from *Dendrobium candidum* was involved in anthocyanin biosynthesis in *D. candidum* stems, through regulation of the expression of *DcF3′H* and *DcUFGT* [55]. The bHLH TF AcB2 has been found to regulate anthocyanin biosynthesis in onion (*Allium cepa* L.) [51]. Luo *et al*. reported that *CsbHLH62* negatively regulates EGCG3″Me biosynthesis in *C. sinensis* [52]. Furthermore, *SlAN11* in tomato has been associated with the regulation of flavonoid biosynthesis through its interaction with bHLH proteins [53]. This study uncovered a novel function of the bHLH TF, revealing that CsbHLH1 plays a role in reducing caffeine levels in ‘Jianghua Kucha’ varieties, including KC3H and XH3H (Fig. 7G and H). An intriguing correlation was found between the expression pattern of *CsbHLH1* and caffeine content when analyzing the major purine alkaloid content of five tea cultivars (Figs 1G and4B), suggesting that CsbHLH1 could act as a negative regulator of caffeine content.

In order to verify the function of *CsbHLH1*, we inhibited the expression of *CsbHLH1* using As ODN technology. This suppression of *CsbHLH1* led to an increase in caffeine level in the leaves of the tea plant (Fig. 7G). The As ODN method involves utilization of synthetic or constructed antisense expression vectors for the expression of oligonucleotide fragments [56]. These fragments, based on the principle of base complementarity, interfere with various aspects of gene functions, including replication, transcription, and mRNA splicing, processing, export, and translation. This interference can effectively regulate cellular processes such as growth and differentiation [56]. This technique is unique in that it focuses on the transient silencing of genes to study their function in plants [57], which can compensate to some extent for the shortcomings of other technologies by not altering plant traits when selecting for transgenic plants compared with marker genes, or by silencing genes in plants where transgenic challenges exist, such as tea plants [58]. The efficacy of As ODN approaches in transiently suppressing gene expression is well documented. CsFtsH5-AsODN plants have shown significantly higher relative electrolyte leakage values than CsFtsH5-AsODN plants under freezing stress, indicating increased cold sensitivity in tea plants as a result of downregulation of *CsFtsH5* [59]. In our research, we observed that lowering the expression of *CsbHLH1* directly led to an increase in both *TCS1* expression and caffeine biosynthesis (Fig. 7G and H). This finding solidifies the conclusion that CsbHLH1 acts as a negative regulator of *TCS1*.

**Figure 8:**
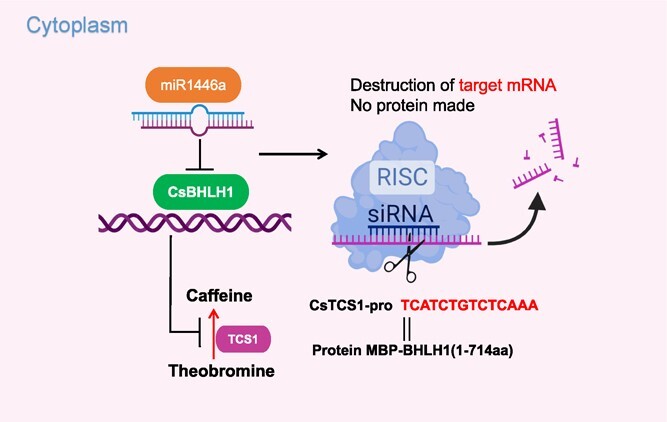
A possible model for the involvement of CsbHLH1 and miR1446 in the biosynthetic regulation of tea plant caffeine.

Additionally, this study delved into the interaction between CsbHLH1 and *TCS1*. The EMSA result showed that CsbHLH1 specifically binds to the MYC binding site in the promoter region of *TCS1* (Fig. 7E). CsbHLH1 directly targets *TCS1* to reduce its expression, which in turn influences caffeine biosynthesis in the tea plant. We transiently co-expressed the reporter (promoter) and effector (TF) by *Agrobacterium*-mediated transformation of tobacco leaves. The LUC/REN ratio of 0.079 indicated that the CsbHLH1 protein inhibits the transcription of *TCS1* (Fig. 7B). Gene expression correlation analysis, gene function validation, and interaction analysis confirmed that CsbHLH1 is a TF of *TCS1*. Previous research has identified CsMYB184, CsNAC7, and CsS40 as TFs that regulate *TCS1*, suggesting that multiple TFs may simultaneously influence the regulation of the same gene [22–24]. This is corroborated by the fact that multiple TFs regulate transcription of the *rpoE* gene in *Escherichia coli* [60]. This was verified by the fact that two TFs, TaPpm1 and TaPpb1, co-regulate anthocyanin biosynthesis in purple pericarps of wheat [61].

While previous research has highlighted the significant role of bHLH TFs in plant metabolite production, the exact regulatory mechanisms of these bHLH factors remain largely unknown. The miRNA sequencing in our study revealed that miR1446a targets and cleaves *CsbHLH1*, thereby regulating the transcription of *TCS1* (Supplementary Data Fg. S5), which leads to increased caffeine accumulation. The miRNAs, as key regulators in plants, significantly contribute to the orchestration of plant secondary metabolism pathways [62, 63], which influences the biosynthesis of secondary metabolites in plants mainly through two pathways. Firstly, miRNAs act directly on genes related to secondary metabolism pathways to cleave target genes and negatively regulate the accumulation of secondary metabolites. Secondly, miRNAs act on transcriptional regulators, further impacting plant secondary metabolism [64, 65]. miRNAs in *Arabidopsis* were found to target *MYB114* and *PAP2*, playing a significant role in the biosynthesis of anthocyanin [65, 66]. *Arabidopsis* miRNAs can also cleave the target gene *SPL* (Squamosa promoter binding protein-like), a negative regulator of anthocyanin biosynthesis, thereby disrupting the WD40-bHLH-MYB complex. By targeting and cleaving the SPL, miR156 promotes anthocyanin accumulation [67]. Conversely, a reduction in miR156 expression bolsters anthocyanin accumulation [67]. Our finding implied a novel role for miR1446a in the regulation of caffeine biosynthesis in plants.

## Conclusion

In conclusion, our findings indicate that the CsbHLH1 TF binds to the promoter region of *TCS1*, specifically between −971 and −1019 bp, effectively repressing the transcriptional activity of *TCS1*. Furthermore, we demonstrated that As ODN_CsbHLH1 effectively represses the expression of the *CsbHLH1*, which subsequently leads to an increase in caffeine content. In addition, we discovered that miR1446a precisely targets and cleaves *CsbHLH1*, consequently suppressing its expression and subsequently influencing caffeine biosynthesis in the tea plant. Based on these results, we propose a possible regulation model (Fig. 8) of caffeine in the tea plant, which provides new insights into the regulation of caffeine biosynthesis and lays the foundation for breeding tea plants with low or high caffeine content. Despite our progress, numerous questions remain about the regulatory mechanisms of caffeine biosynthesis. For instance, it is unclear whether allelic variations in *CsbHLH1* correlate with the expression of *TCS1* or other key genes involved in caffeine biosynthesis. Additionally, understanding how CsbHLH1 cooperates with other reported TFs of *TCS1* to regulate caffeine production in tea plants requires further exploration.

## Materials and methods

### Tea plant materials

Five tea plant cultivars, namely KC3H, XH3H, BXZ, FDDB, and YSXL, were used as experimental materials. XH3H and KC3H belong to ‘Jianghua Kucha’, FDDB originated in Southeast China's Fujian Province, YSXL in Southwest China’s Guangxi Province, and BXZ in central China’s Hunan Province. The five tea cultivars were collected from the tea gardens of the Tea Research Institute of the Hunan Academy of Agricultural Sciences in Changsha, Hunan Province, China. One bud and one leaf of healthy tea plants (KC3H, XH3H, BXZ, FDDB, and YSXL) were collected in spring (April 2021) from 8:30 to 11:00 a.m. Freshly collected samples were stored in liquid nitrogen at −80°C for transcriptome, metabolome, and small RNA analysis and subsequent experiments. Three biological replicates of each cultivar were used for each of the above samples.

### Sample preparation for alkaloid determination

Samples were lyophilized and ground to a fine powder. About 10 mg of tea sample was weighed into a 2-ml centrifuge tube, and 0.5 ml of 70% hot methanol was added. Samples were treated for 10 min in a 70°C water bath. Samples were then centrifuged at 12 000 rpm for 10 min. The supernatant was used for analysis by LC–MS/MS.

### LC–MS/MS analysis

Analyses were performed on a Shimadzu Nexera X2 LC-30 AD high-performance LC system (Kyoto, Japan). The separations were carried out on an Acquity BEH C18 column (2.1 × 100 mm) with 1.7-μm particles from Waters (Milford, MA, USA), which was maintained at 40°C throughout the separation. The optimum gradient used A (0.1% formylic acid (FA) in water)–B (0.1% FA in acetonitrile) as eluent. The gradient conditions are summarized in Supplementary Data Table S12. The injection volume was 1 μl and the flow rate was 0.3 ml/min.

### Mass spectrometry conditions

Detection was carried out using an AB SCIEX 5500 series ion trap, which was equipped with an ESI source and operated in the positive ion mode. The source temperature was 550°C, ion source gas 1 (GS1) 55, ion source gas 2 (GS2) 55, curtain gas (CUR) 35, and ion spray voltage (IS) 5500 V. Detection of metabolites was performed in a positive multiple reaction monitoring (MRM) mode.

### Transcriptome sequencing, RNA preparation, and qRT–PCR

Fifteen cDNA libraries were constructed using total RNA from BXZ, FDDB, XH3H, YSXL, and KC3H. Transcriptome sequencing was performed on triplicate samples, which was carried out by BGI Genomics (Shenzhen, China) using the DNBSEQ platform. FPKM values of key differential genes were analyzed in this study.

RNA preparation and qRT–PCR were performed according to previously described methodology with minor modifications [68]. Total RNA from the tea samples was isolated and purified using the RNAprep Pure Plant Kit (Tiangen Biotech, Beijing, China). Subsequently, cDNA was reverse-transcribed using a One-Step gDNA Removal kit (TransGen Biotech, Beijing, China) and was then used as template for qRT–PCR according to the Two-Step qRT-PCR Kit instructions (TransGen Biotech, Beijing, China), with the internal reference gene *GAPDH*. The analysis was performed using 2^−ΔΔCT^, and qRT–PCR primers are listed in Supplementary Data Table S9 [69].

### Small RNA library construction and sequencing

Small RNA sequencing was performed by BGI Genomics on triplicate samples using the Solexa platform. The cleaned target sequences were classified and annotated by GenBank and Rfam database comparison, and the statistics of sequence length distribution as well as the statistics of public sequences among samples and the types and numbers of unique sequences were determined. Sequences were aligned to miRBase 21.0 using the nucleotide–nucleotide Basic Local Alignment Search Tool (BLASTn) to identify known conserved miRNAs. To identify new miRNAs we used the Mireap program (http://sourceforge.net/projects/mireap). Finally, the corresponding identified known miRNAs and new miRNAs were subjected to target gene prediction and functional analysis.

### Subcellular localization of CsbHLH1

A bacterial broth containing 35S::CsbHLH1-GFP was injected into the leaves of tobacco plants and the leaves that were injected were used as the experimental group. At the same time, tobacco leaves were injected with pSuper1300-GFP empty vector as a control group. The injected tobacco plants were incubated for 36 h (23 ± 2°C, 85% relative humidity) and their leaves were cut into square pieces (2 mm × 2 mm) at the injection site [70]. The results of the experiments were observed using a laser scanning confocal microscope (LSCM; Nikon Corporation, Tokyo, Japan).

### Dual-luciferase reporter assays

The dual-report vector included the smallest TATA region of firefly luciferase (LUC) driven by five GAL4 binding elements (5 × GAL4) and CaMV 35S. *Renilla* luciferase (REN) driven by the 35S promoter was used as the control. The full-length cDNA of *CsbHLH1* was cloned into pGreenII 62-SK vector, and the recombinant plasmid pGreenII 62-SK-CsbHLH1 was constructed as effector. At the same time, the promoter sequence of *TCS1* was cloned into pGreenII 0800-LUC dual reporter vector as the reporter. Next, these plasmids were introduced into *Agrobacterium tumefaciens* for transient expression and the preparation was cultured for 48–72 h [71]. The Dual-Luciferase^®^ Reporter Assay System kit was used to determine the ratio of LUC to REN luciferin in tobacco leaves.

### Yeast one-hybrid assays

The full-length sequence of *CsbHLH1* was linked to the yeast expression vector pGADT7, leading to creation of recombinant plasmid pGADT7-CsbHLH1 (1–714 aa). Simultaneously, the promoter sequence of *TCS1* (−953 to −1556 bp) was ligated into the pHis2 vector, resulting in the recombinant plasmid pHis2-TCS1. The pHis2-TCS1 and pGADT7-CsbHLH1 plasmids were mixed as the experimental group. The pHis2-TCS1 and empty pGADT7 plasmids were mixed as the self-activating group. The yeast strain Y187 was then transformed with these plasmids. The yeast cells were then inoculated on triple-deficient (SD/−His/−Leu/−Trp) medium with or without 3-AT in serial dilutions and incubated at 28°C for 3 days [72]. The formation of white colonies in the experimental group and lack thereof in the self-activation group would suggest successful transactivation.

### Electrophoretic mobility shift assay

The pMAI-C6T-CsbHLH1 fusion construct was formed by inserting the full-length *CsbHLH1* sequence into the pMAI-C6T expression vector. The recombinant plasmid was inserted into BL21 receptor cells and the addition of 0.7 mM IPTG induced protein expression [73]. Protein purification was then carried out using the Ni-NTA Sefinose Resin Kit (Sangon Biotech, Shanghai, China).

For the biosynthesis of the universal probe, a 102-bp DNA fragment containing the promoter region of an MYC element at the 5′ end of *TCS1* was used. The sequence of the competing probes was identical to that of the universal probe. The mutant probe was synthesized by replacing the sequence of the MYC site (CATGTG) with the base A, while retaining the rest of the sequence identical to the universal probe [74]. DNA probes for EMSA were prepared at Wuhan Jinkairui Bioengineering Co. Ltd (Wuhan, China). Probes and samples were loaded on 6% polyacrylamide gels and run in TBE buffer.

### Antisense oligonucleotide experiment

Several optimal As ODNs were designed and selected based on the sequences of the tea plant gene *CsbHLH1* using the online software Soligo [75] (http://sfold.wadsworth.org/cgi-bin/index.pl). Tea plant shoots (a bud and two leaves) were placed in a centrifuge tube with 20 μM CsbHLH1-related As ODNs (placed in water as control). Samples were taken after 6, 24, and 48 h to detect changes in *CsbHLH1* gene expression and caffeine content.

### Validation of cleavage sites using modified RLM–RACE

A quantity of 1 μl from each of the qualified RNA samples was mixed, and was then used for modified RLM–RACE reverse transcription. The GeneRacer kit (Invitrogen, USA) was used for RLM–RACE following the specific test procedure described elsewhere [76]. The target gene cleavage primers were designed using DNAMAN and nested PCR amplification was performed using specific and universal primers (Supplementary Data Table S3). TA cloning of these isolated products was performed, followed by the selection of multiple positive single colonies for sequencing. Sequencing was carried out by T&J Bioengineering (Shanghai) Co. Ltd.

## Acknowledgements

This work was supported by the Natural Science Foundation of Hunan Province (2021JC0007), the National Natural Science Foundation of China (U22A20500, U19A2030, 32172629), and the key Science and Technology Research Projects of Hunan Province (2021NK0008, 2021NK1020).

## Author contributions

Q.J. designed and performed the experiments and drafted the paper. Z.W. analyzed the data and prepared the figures and tables. DS performed data analysis and reviewed and revised the manuscript. L.C., C.S., F.S., S.X., F.H., Z.C., X.Z., J.H., G.L., Q.S., and M.H. participated in the experiments and data analysis. Z.L., J.H., N.T., and S.L. conceived the study, participated in the coordination, data analysis, and interpretation, and drafted and reviewed the manuscript. All authors read and approved the final manuscript.

## Data availability statement

All the data supporting our findings are contained within the manuscript. All raw transcriptome data reported in this article have been deposited in the Sequence Read Archive (SRA) under accession number PRJNA986690 and PRJNA1056246.

## Conflict of interests

The authors declare they have no competing interests.

## Supplementary information

Supplementary data is available at *Horticulture Research* online.

## Supplementary Material

Web_Material_uhad282
