# Supplementary material for: mRNA-miRNA analyses reveal the involvement of CsbHLH1 and miR1446a in the regulation of caffeine biosynthesis in Camellia sinensis
Source: Hortic Res. 2023 Dec 29;11(2):uhad282. doi: 10.1093/hr/uhad282 (PMC11648165; doi:10.1093/hr/uhad282)
Supplement: Web_Material_uhad282 [file web_material_uhad282.zip › Supplementary Figure.docx]

**Figure S1.** **The main pathways of alkaloid biosynthesis and catabolism and its regulation of caffeine biosynthesis in tea plants.** Anase, adenosine nucleosidase; ADK, Adenosine kinase; 5' -deaminase; IMP, inosine 5' -monophosphate; IMPDH, IMP dehydrogenase; XMP, xanthosine 5' - monophosphate; GMP, guanosine 5' -monophosphate; GMPS, GMP synthase; GK, guanosine kinase; GDA, guanine deaminase; 7-NMT, 7-methylxanthosine synthase; N-MeNase, N-methyl nucleosidase; 3-NMT, theobromine synthases; 1-NMT/TCS1, caffeine synthases; 9-NMT, theacrine synthases; 7-NDM, 7-N- demethylase; 1-NDM, 1-N-demethylase; 3-NDM, 1-N-demethylase; XO, Xanthine dehydrogenase; UOX, Uricase; ALN, Allantoin enzyme; ALLC, Allantoic acidase; URE, Urine enzyme.


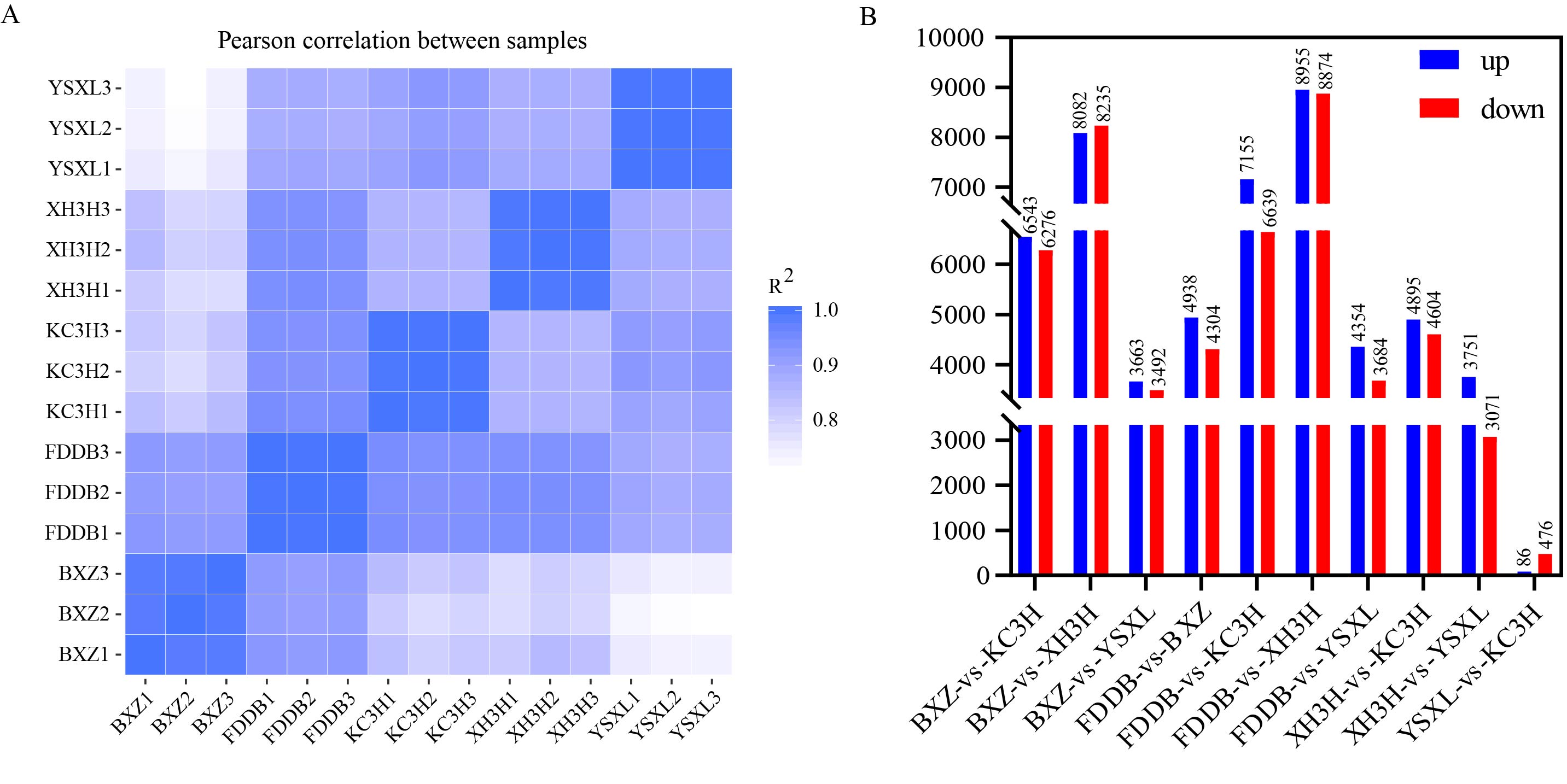


**Figure S2.** **Expression patterns of RNA-seq data for BXZ, FDDB, KC3H, XH3H, and YSXL samples.** (A) Heatmap of the correlation analysis of the gene expression levels between each pair of samples. (B) Upregulated and downregulated DEGs number in different compared groups.


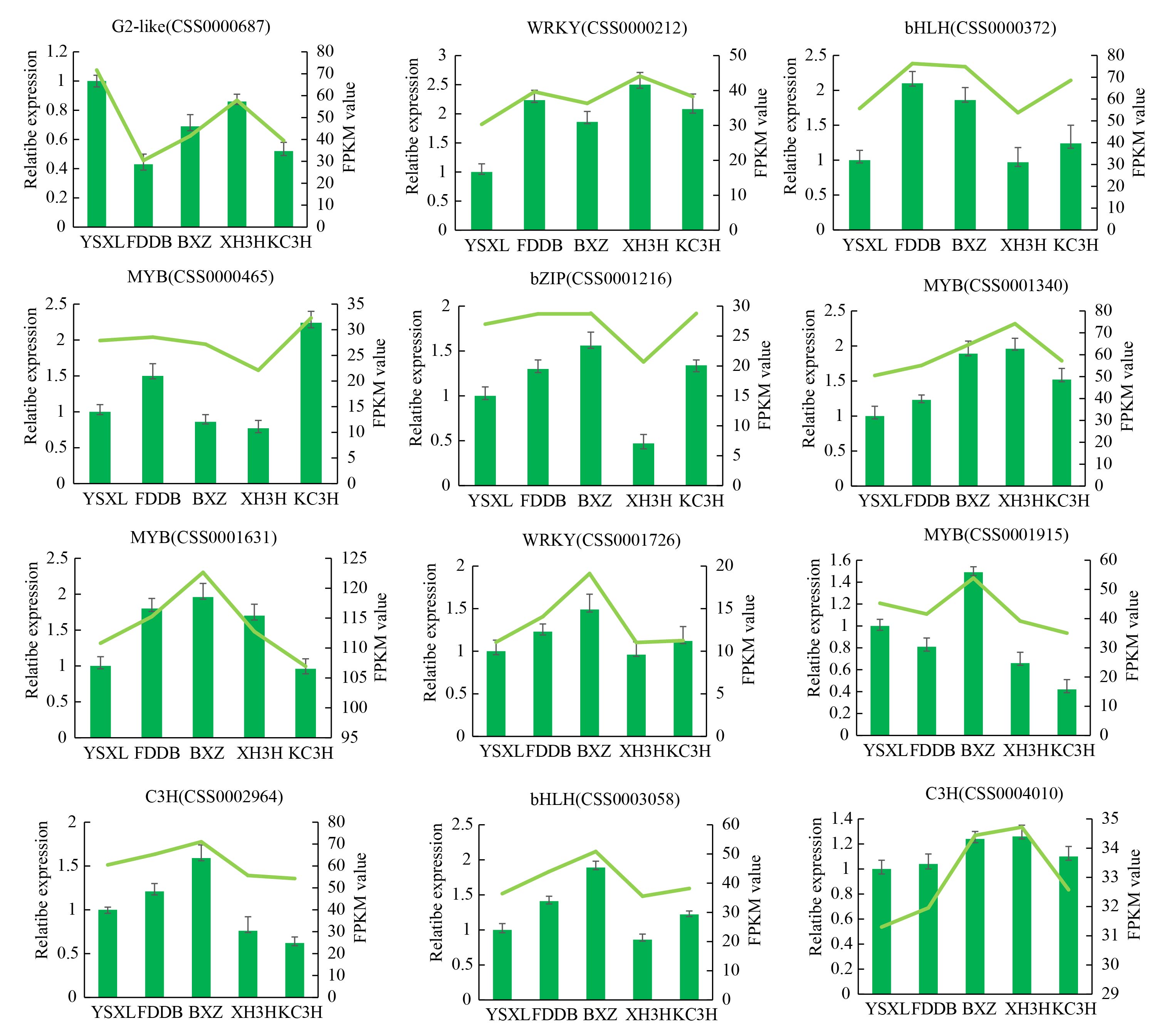


**Figure S3.** **Validation of quantitative RT-PCR.**


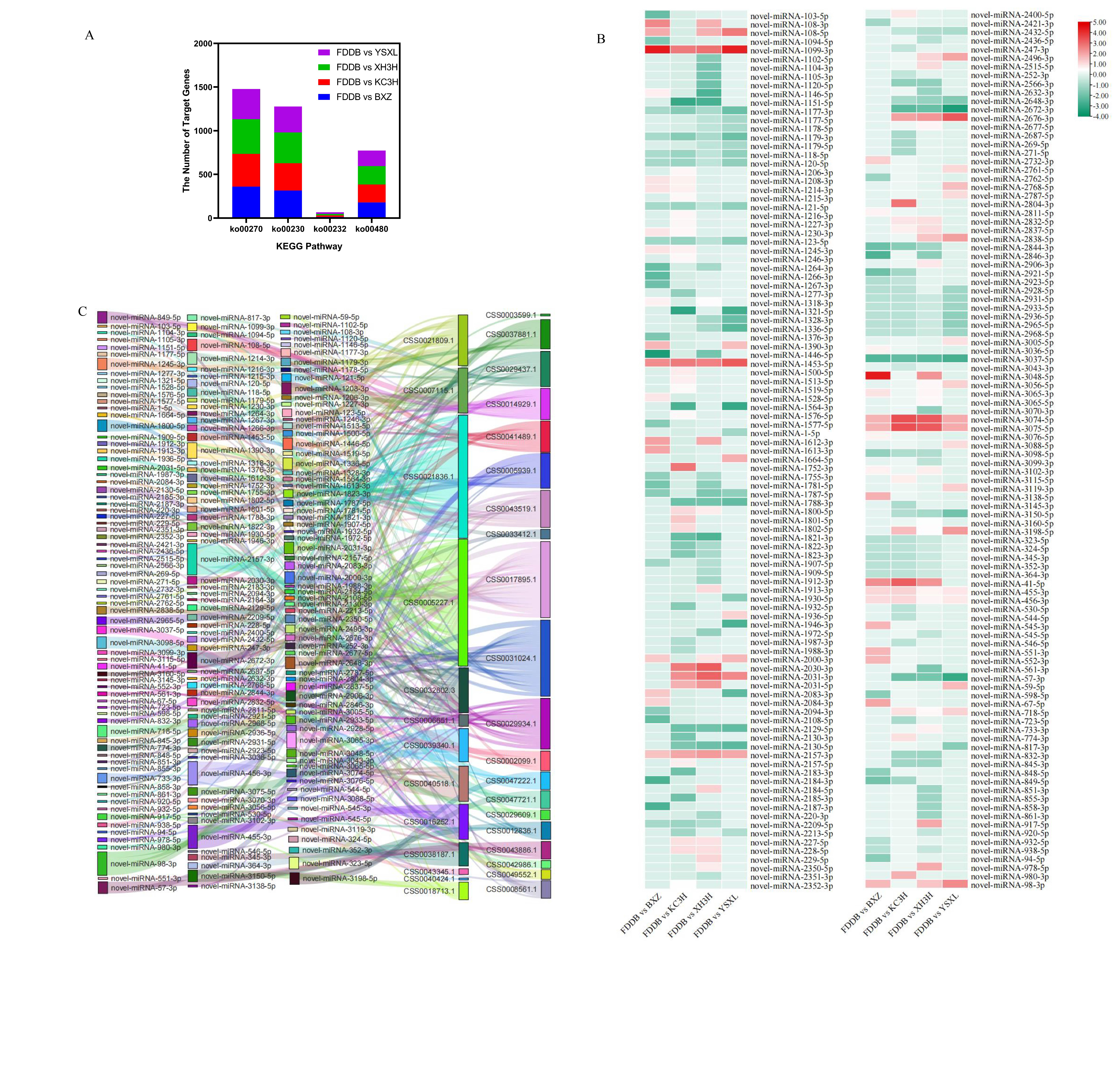


**Figure S4．Analysis of the interaction between miRNAs and their target genes.** (A) Four bar graphs consisting of purple, green, red, and blue, representing FDDB vs YSXL, FDDB vs XH3H, FDDB vs KC3H, and FDDB vs BXZ, respectively, on which the number of target genes is indicated. The abscissa shows the KEGG pathways, named cysteine and methionine metabolism (ko00270), purine metabolism (ko00230), caffeine metabolism (ko00232), and glutathione metabolism (ko00480). (B) Regulation of differentially expressed miRNAs in the four comparison groups. The abscissa represents the groups compared and the ordinate represents the miRNAs that were differentially expressed. Panel (C) shows the interaction between miRNAs and target genes.


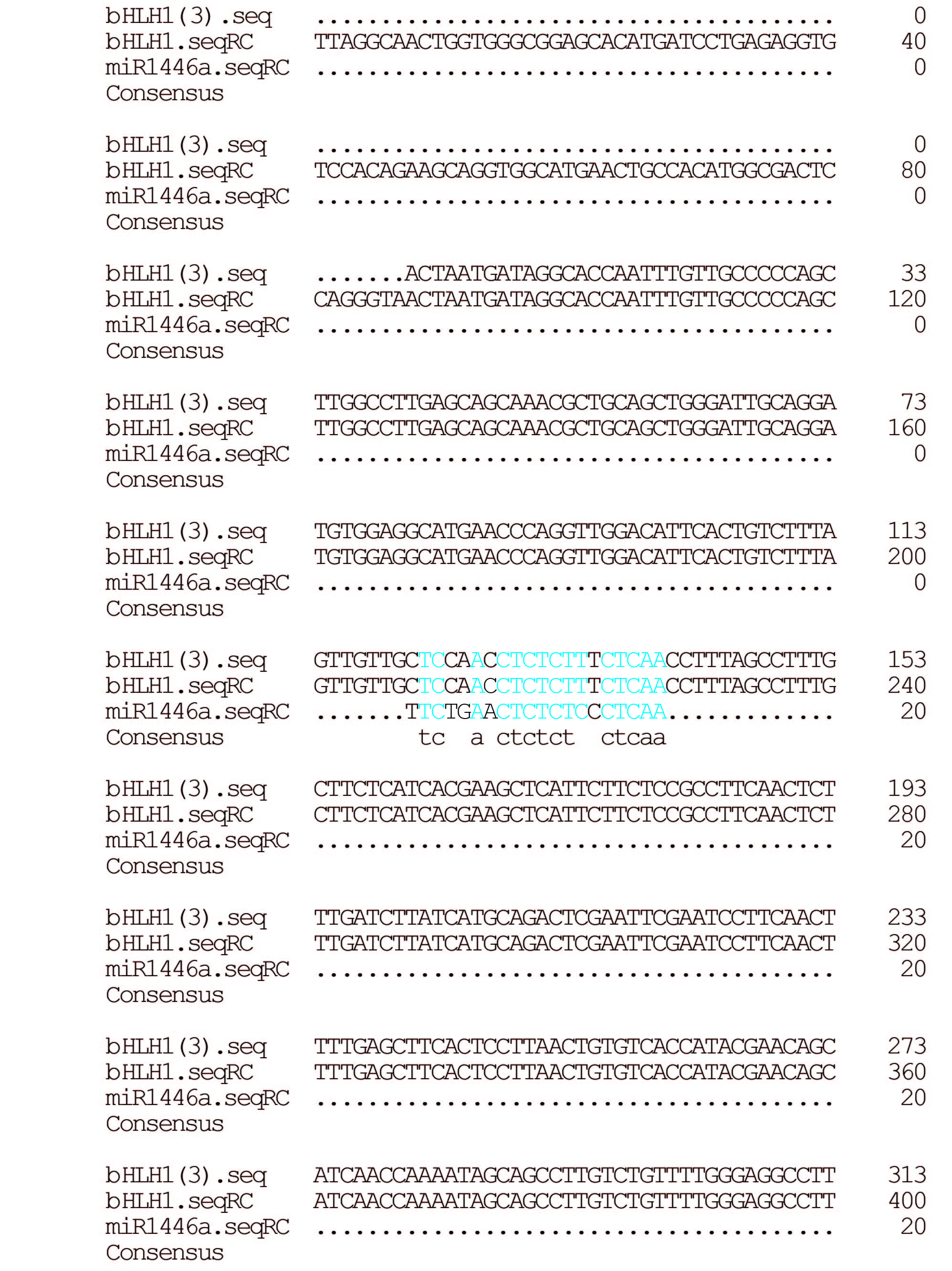


**Figure S5.** **The cleavage site was verified by 5'RLM-RACE.** The miR1446a targets the cleavage of the downstream transcription factor CsbHLH1, starting at the 88bp position. bHLH1(3) is a sequenced sequence.
